# KIR-HLA gene diversities and susceptibility to lung cancer

**DOI:** 10.1038/s41598-022-21062-1

**Published:** 2022-10-14

**Authors:** Marjan Hematian Larki, Elham Ashouri, Shaghik Barani, Seiyed Mohammad Ali Ghayumi, Abbas Ghaderi, Raja Rajalingam

**Affiliations:** 1 Shiraz Institute for Cancer Research, School of Medicine, Shiraz University of Medical Sciences, Shiraz, Iran; 2 Department of Internal Medicine, School of Medicine, Shiraz University of Medical Sciences, Shiraz, Iran; 3 Department of Immunology, School of Medicine, Shiraz University of Medical Sciences, Shiraz, Iran; 4 Immunogenetics and Transplantation Laboratory, Department of Surgery, University of California San Francisco, San Francisco, CA USA

**Keywords:** Cancer, Immunology

## Abstract

Killer-cell immunoglobulin-like receptors (KIR) are essential for acquiring natural killer (NK) cell effector function, which is modulated by a balance between the net input of signals derived from inhibitory and activating receptors through engagement by human leukocyte antigen (HLA) class I ligands. KIR and HLA loci are polygenic and polymorphic and exhibit substantial variation between individuals and populations. We attempted to investigate the contribution of *KIR* complex and *HLA class I* ligands to the genetic predisposition to lung cancer in the native population of southern Iran. We genotyped 16 *KIR* genes for a total of 232 patients with lung cancer and 448 healthy controls (HC), among which 85 patients and 178 HCs were taken into account for evaluating combined *KIR*-*HLA* associations. *KIR2DL2* and *2DS2* were increased significantly in patients than in controls, individually (OR 1.63, and OR 1.42, respectively) and in combination with *HLA-C1* ligands (OR 1.99, and OR 1.93, respectively). *KIR3DS1* (OR 0.67) and *2DS1* (OR 0.69) were more likely presented in controls in the absence of their relative ligands. The incidence of CxTx subset was increased in lung cancer patients (OR 1.83), and disease risk strikingly increased by more than fivefold among genotype ID19 carriers (a CxTx genotype that carries *2DL2* in the absence of *2DS2*, OR 5.92). We found that genotypes with *iKIRs* > *aKIRs* (OR 1.67) were more frequently presented in lung cancer patients. Additionally, patients with lung cancer were more likely to carry the combination of CxTx/*2DS2* compared to controls (OR 2.04), and *iKIRs* > *aKIRs* genotypes in the presence of *2DL2* (OR 2.05) increased the likelihood of lung cancer development. Here we report new susceptibility factors and the contribution of *KIR* and *HLA-I* encoding genes to lung cancer risk, highlighting an array of genetic effects and disease setting which regulates NK cell responsiveness. Our results suggest that inherited *KIR* genes and *HLA-I* ligands specifying the educational state of NK cells can modify lung cancer risk.

## Introduction

Lung cancer, the most common cause of cancer-related mortality worldwide is generally classified into main histological subtypes, including non-small cell lung cancer (NSCLC) and small cell lung cancer (SCLC)^1,2^. Lung mucosa is constantly exposed to inhaled environmental pollution, dust, smoke, and pathogens; hence a dynamic network of tissue-resident immune cells continually keeps monitoring the lung to maintain tissue homeostasis^3^. The immune system also has a vital role in cancer initiation and progression. Natural killer cells (NK cells) are part of innate immune cells that serve as a first-line of defense with the capacity to eliminate virally infected cells and neoplastic transformations without prior sensitization^4^. These cytotoxic cells mediate antitumor responses by exploiting death receptors, releasing pro-inflammatory cytokines and perforin/granzyme granules exocytosis^5^.

Interestingly, over 10% of resident lymphocytes in the lung are NK cells^6^. The association of NK cell dysfunction with tumor progression was revealed in KRAS-driven lung cancer, in which NK cells protect tumors at the early stage but cannot prevent tumor progression^7^. Also, it is shown that resident NK cells have a pivotal role in resistance to experimental lung metastasis by producing IFN-γ^8^. Although the infiltration of NK cells was indicated as a favorable prognostic factor in lung cancer^9,10^, the functional reactivity of intratumoral NK cells is crucial in addition to the degree of NK cell infiltration. It has been demonstrated that NK cell dysfunction at the late stage is caused by the suppressive tumor microenvironment (TME)^7^. NK cells isolated from NSCLC patients, which depict different expression patterns, have impaired interferon-γ (IFN-γ) production capability and lower cytotoxicity than non-tumoral NK cells^11–13^.

The activity of NK cells is modulated by a balance between the net input of signals derived from an array of inhibitory and activating receptors^14^. Among these germ-line encoded receptors, killer-cell immunoglobulin-like receptors (KIR) are essential for acquiring NK cell effector function^15,16^. KIRs are encoded by a cluster of polymorphic and homologous genes located at chromosomal region 19q13.4^17^. KIR region comprises activating (*2DS1*-*5*, *3DS1*) and inhibitory (*2DL1*-*3*, *2DL5A*/*B*, *3DL1*) genes, divided by framework KIRs into centromeric (*3DL3* to *3DP1*) and telomeric (*2DL4* to *3DL2*) intervals^18^. Although (5′) centromeric and (3′) telomeric regions contain various combinations of activating (*aKIR*) and inhibitory (*iKIR*) genes, certain adjacent KIRs tend to be strongly linked^17,19^. Considering strong linkage disequilibrium (LD) among *KIR* genes, it is difficult to isolate each *KIR* gene***'***s effect on NK cell response^20^. Based on gene content within both intervals, the KIR locus segregates in distinct haplotypes, mainly known as A (fixed combination of genes, mostly *iKIRs*) and B (variable number of *iKIRs*/*aKIRs*)^17,21^. The KIR gene cluster displays tremendous variation due to gene-content diversity and haplotypic variety^17^. In addition to gene content diversity, allelic polymorphism extends KIR variations and accounts for differential expression levels of KIRs on the surface of NK cells^22,23^.

The classical HLA class I molecules (HLA-A, B, C) as major KIR ligands have been categorized into 4 types of KIR-binding epitopes (C1, C2, Bw4, A3/A11) according to amino acid sequences^24^. KIR2DL1 and KIR2DL2/3 mainly bind to a group of ligands encoded by HLA-C alleles that differed by Lys/ Asn dimorphism at position 80 (HLA-C2 and HLA-C1, respectively)^25^. KIR3DL1 was found to interact with HLA-Bw4, and KIR3DL2 recognizes HLA-A3/A11^26,27^. The Ile/Thr dimorphism at position 80 defines the affinity of KIR binding to the Bw4 motif, in which Ile80 exhibits a greater affinity for 3DL1^28^. The growing understanding of activating KIR/ HLA interactions indicates that activating KIR3DS1, 2DS1, and 2DS2 bind to the same HLA class I with lower affinity than their homologous inhibitory counterparts^29–32^. However, other KIR/HLA pair interactions haven’t been distinctly demonstrated. It is shown that different iKIRs exhibit various binding affinities for HLA-I ligands. Interestingly the peptide presented by HLA subtypes seems to play a role in binding affinity alteration^33^. Signals derived from iKIRs interacting with self-HLA ligands set a threshold of activation for NK cells leading to a maturation process titled “education” or “licensing”^34,35^. Besides development and self-tolerance, education renders NK cells capacity to recognize diseased cells with downregulated or lacking HLA class I expression, referring to the “missing self” hypothesis^34^. Lacking a specific HLA-I ligand or any responding iKIR, yields hypo-responsive NK clones with high activation threshold^30^. It is particularly noteworthy that *KIR*-gene complex diversity influences surface expression, ligand specificities, ligand binding affinity, and subsequent signal transduction through KIR-HLA class I interaction^36–38^. Such an intense heterogeneity in *KIR* and *HLA* complex prominently affects NK cell responses and is associated with disease susceptibility^39^. Genome-wide association studies (GWAS) have identified 45 loci associated with lung cancer risk^40^. More specifically, SNPs from 5p15.33, 6p21.33, and 15q25.1 regions are strongly associated with lung cancer in Caucasians^41^. However, the composition of multiple highly-homogeneous gene content, intense polymorphism, strong linkage disequilibrium between multiple loci, and low/no coverage by GWAS reagents pose challenges to studying the polymorphism of KIR (19q13) and HLA (6p21) gene families by GWAS.

The present study aimed to investigate the contribution of *KIR* complex and *HLA-I* ligands to the genetic predisposition to lung cancer in the native population of Fars province, located in the southern part of Iran, and to disclose possible associations with the dysfunctional state of NK cells in the context of lung cancer (Table 1).

**Table 1:** Characteristics of the study population.

Characteristics	Lung cancer patients (n = 232)	Healthy controls (n = 448)
Mean age ± SD	64.4 ± 11.1	58.13 ± 12.66
**Gender**		
Female	34 (14.7%)	150 (33.5%)
Male	198 (85.3%)	298 (66.5%)
**Smoking status**		
Smoker	51 (21.98%)	64 (14.3%)
Non-smoker	0 (0.0%)	3 (0.7%)
Unknown	181 (78.02%)	381 (85.0%)
**Lung cancer subtypes**		
SCLC	53 (22.8%)	
NSCLC	179 (77.2%)	
Squamous cell carcinoma	144 (62.1%)	
Adenocarcinoma	35 (15.1%)	

## Results

### Susceptibility/resistance influence of specific B haplotype-associated KIRs on lung cancer risk

The distribution of 16 KIR genes were determined in patients and HCs (Table 2). As observed, framework genes (*3DL3*, *3DP1*, *2DL4*, *3DL2*) were presented in all subjects. Two adjacent B haplotype-associated genes *2DL2* (67.2% vs. 55.4%, *p* = 0.003, OR 1.63, CI 1.17–2.26) and *2DS2* (62.9% vs. 54.6%, *p* = 0.041, OR 1.42, CI 1.02–1.96) were significantly increased in patients in comparison with controls. Activating genes *2DS1* (50.2% vs. 41.4%, *p* = 0.029, OR 0.69, CI 0.5–0.95) and *3DS1* (47.3% vs. 37.5%, *p* = 0.015, OR 0.67, CI 0.48–0.92) were more frequently presented in controls than patients, conferring protection against the lung cancer. Table 2 also shows the results with further assessment of *KIR* genes and their associations with lung cancer subtypes (NSCLC, SCLC).

**Table 2:** KIR gene frequencies among patients with lung cancer and healthy controls.

*KIR* genes	Healthy controls	Lung cancer			Comparisons					
		Lung cancer	NSCLC	SCLC	Lung cancer versus HC		NSCLC versus HC		SCLC versus HC	
	n = 448	n = 232	n = 179	n = 53	*p* value	OR (95% CI)	*p* value	OR (95% CI)	*p* value	OR (95% CI)
	%F (N + /n)	%F (N + /n)	%F (N + /n)	%F (N + /n)						
**Group-A haplotype-associated *KIR* genes**										
*2DL1*	98.0 (439/448)	97.4 (226/232)	97.2 (174/179)	98.1 (52/53)						
*2DL3*	88.2 (395/448)	85.3 (198/232)	84.4 (151/179)	88.6 (47/53)						
*3DL1*	92.6 (415/448)	95.3 (221/232)	96.6 (173/179)	92.4 (49/53)						
*2DS4*	92.9 (416/448)	94.8 (220/232)	95.5 (171/179)	92.4 (49/53)						
*2DS4fl*	12.9 (31/242)	10.4 (18/174)	10.2 (14/137)	10.8 (4/37)						
*2DS4del*	73.5 (178/242)	70.6 (123/174)	70.8 (97/137)	70.3 (26/37)						
*2DS4fl,del*	13.6 (33/242)	19.0 (33/174)	19.0 (26/137)	18.9 (7/37)						
**Group-B haplotype-associated *KIR* genes**										
*2DL2*	55.4 (248/448)	67.2 (156/232)	65.9 (118/179)	71.6 (38/53)	0.003	1.63 (1.17–2.26)	0.016	1.56 (1.087–2.24)	0.027	2.04 (1.09–3.82)
*2DL5*	66.1 (296/448)	63.8 (148/232)	64.2 (115/179)	62.2 (33/53)						
*3DS1*	47.3 (212/448)	37.5 (87/232)	36.8 (66/179)	39.6 (21/53)	0.015	0.67 (0.48–0.92)	0.02	0.65 (0.43–0.93)		
*2DS1*	50.2 (225/448)	41.4 (96/232)	41.3 (74/179)	41.5 (22/53)	0.029	0.69 (0.5–0.95)				
*2DS2*	54.5 (244/448)	62.9 (146/232)	62.0 (111/179)	66.0 (35/53)	0.041	1.42 (1.02–1.96)				
*2DS3*	41.5 (186/448)	40.1 (93/232)	39.6 (71/179)	41.5 (22/53)						
*2DS5*	39.5 (177/448)	32.8 (76/232)	32.9 (59/179)	32.0 (17/53)						
**Framework genes/pseudogenes**										
*2DL4*	100 (448)	100 (232)	100 (179)	100 (53)						
*3DL2*	100 (448)	100 (232)	100 (179)	100 (53)						
*3DL3*	100 (448)	100 (232)	100 (179)	100 (53)						
*2DP1*	97.8 (438)	96.1 (223)	94.9 (170)	100 (53)						
*3DP1*	100 (448)	100 (232)	100 (179)	100 (53)						

*N*+ number of individuals positive for the gene, *n* number of individuals tested for the gene, *HC* healthy control, *OR* odds ratio, *CI* confidence interval. *p* < 0.05: statistically significant; based on two-tailed Fisher’s exact test.

### Risk-association of KIR-HLA combinations with lung cancer

To evaluate the contribution of *KIR*-*HLA* combinations to lung cancer risk, we analyzed the distribution of *KIR* genes and cognate *HLA-I* ligands within a group of 85 patients along with 178 HCs (Table 3). Similar to the results with individual *KIR* genes, coexistence of *2DL2*/*C1* (55.3% vs. 38.3%, *p* = 0.026, OR 1.99, CI 1.11–3.56) and *2DS2*/*C1* (50.8% vs. 34.7%, *p* = 0.036, OR 1.93, CI 1.08–3.46) were found to occur more frequently in lung cancer patients than controls. A significantly less frequent carriage of *3DL1*-*Bw4* combination was detected in patients with lung cancer than HCs (44.2% vs. 62%, *p* = 0. 014, OR 0.48, CI 0.25–0.91). However, no significant differences were observed between the two groups for the prevalence of *2DS1* and *3DS1* genes combined with respective *HLA-C2* and *HLA-Bw4* ligands, suggesting that particular educational states of NK cells may alter NK cell functionality in the lung cancer setting.

**Table 3:** Frequency of KIR-HLA combinations, and HLA class-I ligands among patients with lung cancer and healthy controls.

KIR/HLA	Healthy controls	Lung cancer	Lung cancer versus HC	
	n = 448	n = 232	*p* value	OR (95% CI)
	%F (N + /n)	F% (N + /n)		
**KIR-binding motif**				
HLA-C1	74.2 (124/167)	81.5 (53/65)		
HLA-C2	73.6 (123/167)	72.3 (47.65)		
HLA-Bw4	62.1 (95/153)	48.1 (25/52)		
Bw4T80	45.7 (70/153)	44.2 (23/52)		
Bw4I80	16.3 (25/153)	3.8 (2/52)	0.018	0.2 (0.047–0.9)
HLA-A3/A11	39.2 (31/79)	38.1 (24/63)		
HLA-A23/24/25/32	49.3 (39/79)	44.4 (28/63)		
**KIR-HLA combination**				
3DL2 + A3/A11	39.2 (31/79)	38.1 (24/63)		
2DL1 + C2	71.8 (120/167)	72.3 (47/65)		
2DL3 + C1	68.8 (115/167)	69.2 (45/65)		
2DL2 + C1	38.3 (64/167)	55.3 (36/65)	0.026	1.99 (1.11–3.56)
3DL1 + Bw4	62.0 (95/153)	44.2 (23/52)	0.014	0.48 (0.25–0.91)
2DS1 + C2	26.6 (45/169)	27.7 (18/65)		
2DS2 + C1	34.7 (58/167)	50.8 (33/65)	0.036	1.93 (1.08–3.46)
3DS1 + Bw4	20.2 (31/153)	15.3 (8/52)		

*N*+ number of individuals positive for the gene, *n* number of individuals tested for the gene, *HC* healthy control, *OR* odds ratio, *CI* confidence interval. *p* < 0.05: statistically significant; based on two-tailed Fisher’s exact test.

When *HLA-I* ligands were analyzed separately, *HLA-Bw4* (*Ile80*) was less prevalent (3.8% vs. 16.3%, *p* = 0.018, OR 0.2, CI 0.47–0.9) in lung cancer patients than in controls (Table 3). This points to the possibility that the difference in the distribution of *3DL1*-*Bw4* combination could be driven by *Bw4* (*Ile80*)*.* The results of comparing the frequency of remaining *HLA-I* genes didn’t reach the level of statistical significance.

Subgroup analysis wasn’t accomplished regarding the association of *KIR*-*HLA* combinations and *HLA-I* ligands with different lung cancer subtypes owing to the inadequate sample size included in the HLA typing method.

### Susceptibility/resistance influence of specific Bx genotype-associated gene clusters on lung cancer risk

**Figure 1:**
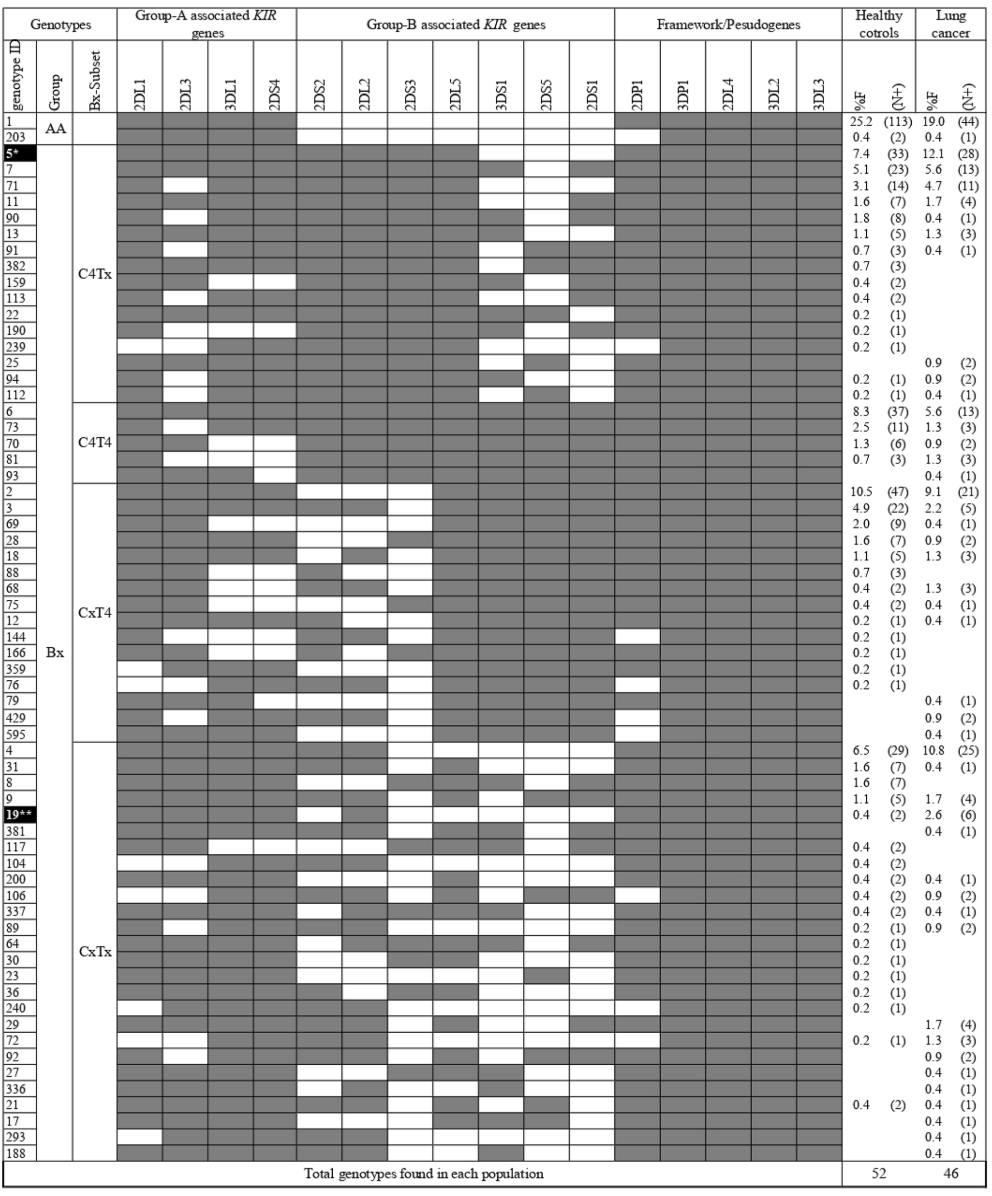
KIR gene content diversity among patients with lung cancer and healthy controls. N+ : number of individuals positive for the gene; n: number of individuals tested for the gene; Gene content of 65 KIR genotypes are displayed by presence/shaded boxes or absence/white boxes of 16 KIR genes. Distribution of genotypes with ID5, and ID19 highlighted by dark boxes were found to be significantly different between lung cancer patients and controls. *p = 0.048, OR 1.73, 95% CI: (1.015–2.93); **p = 0.021, OR 5.92, 95% CI: (1.18– 29.58).

KIR genotype profiles of 232 lung cancer patients and 448 HCs are listed in Table 4. A set of 65 genotypes differentiated by KIR gene content were detected in a total of 680 study participants from southern Iran. Thirty-three genotypes occurred in both patients and controls, 13 genotypes occurred only in patients, and 19 genotypes occurred only in controls.Genotype ID5 (12.1% vs. 7.4%, *p* = 0.048, OR 1.73, CI 1.015–2.93) was significantly more frequent in patients in comparison with controls. Strikingly, the genotype ID19, which is a rare CxTx genotype carrying *2DL2* in the absence of *2DS2* was associated with a more than fivefold increase in lung cancer risk (2.4% vs. 0.4%, *p* = 0.021, OR 5.92, CI 1.18–29.58) (Fig. 1).

**Table 4:** KIR genotype frequencies among patients with lung cancer and healthy controls.

*KIR*	Healthy controls	Lung cancer			Comparisons					
		Lung cancer	NSCLC	SCLC	Lung cancer versus HC		NSCLC versus HC		SCLC versus HC	
	n = 448	n = 232	n = 179	n = 53	*p* value	OR (95% CI)	*p* value	OR (95% CI)	*p* value	OR (95% CI)
	%F (N+)	%F (N+)	%F (N+)	%F (N+)						
**KIR genotypes**										
AA	25.7 (115)	19.4 (45)	19.5 (35)	16.9 (9)						
Bx	74.3 (333)	80.6 (187)	80.4 (144)	83.1 (44)						
CxT4	22.7 (102)	17.6 (41)	18.4 (33)	15.0 (8)						
C4Tx	23.4 (105)	28.4 (66)	29.6 (53)	24.5 (13)						
C4T4	12.7 (57)	9.5 (22)	8.37 (15)	13.2 (7)						
CxTx	15.4 (69)	25.0 (58)	24.0 (43)	30.2 (16)	0.0035	1.83 (1.23–2.71)	0.015	1.74 (1.13–2.66)	0.011	2.37 (1.25–4.5)
C4 Linkage groups	36.2 (162)	37.9 (88)	37.9 (68)	37.7 (20)						
T4 Linkage groups	35.4 (159)	27.1 (63)	26.8 (48)	28.3 (15)	0.03	0.67 (0.47–0.97)	0.0039	0.66 (0.45–0.97)		

*N* + number of individuals positive for the gene, *n* number of individuals tested for the gene, *HC* healthy control, *OR* odds ratio, *CI* confidence interval. *p* < 0.05: statistically significant; based on two-tailed Fisher’s exact test.

The distribution of main genotypes AA and Bx were comparable among the two groups. Significant differences in the frequencies of Bx genotype subsets categorized based on C4 and T4 gene clusters were observed, whereby T4 gene cluster (35.4% vs. 27.1%, *p* = 0.03, OR 0.67, CI 0.47–0.97) was found to be associated with reduced risk of lung cancer in our study population. In contrast, we noticed significantly more frequent CxTx subset (25% vs. 15.4%, *p* = 0.0035, OR 1.83, CI 1.23–2.71) in patients indicating an association between this subset and increased risk of lung cancer (Table 4). KIR genotype frequencies and their statistical associations with lung cancer subtypes (NSCLC, SCLC) are summarized in Table 4.

### Risk-association of co-existing susceptibility factors with lung cancer

To explore further the possible associations of carrying gene contents varied in the number of inhibitory and activating *KIR* genes on susceptibility to lung cancer, we assessed comparisons with regard to different numbers of *iKIRs* and *aKIRs* (Table 5). Carriage of genotypes with *iKIRs* > *4* was more likely presented in patients (75% vs. 67%, *p* = 0.035, *P*_*c*_ = 0.14, OR 1.48, CI 1.036–2.11), on the contrary genotypes with *aKIRs* > 4 were more frequently found in HCs (50% vs. 40.1%, *p* = 0.015, *P*_*c*_ = 0.06, OR 0.66, CI 0.48–0.92). Lung cancer patients were more likely to carry genotypes with *iKIRs* > *aKIRs* (64.7% vs. 52.2%, *p* = 0.002, *P*_*c*_ = 0.008, OR 1.67, CI 1.2–2.32), and this difference remained significant after being corrected for multiple comparisons, suggesting the strong association of genotypes with *iKIRs* > *aKIRs* with susceptibility to lung cancer.

**Table 5:** Carrier frequency of various susceptibility-related factor combinations among patients with lung cancer and healthy controls.

KIR combination	Healthy controls	Lung cancer	Lung cancer versus HC		
	n = 448	n = 232	*p* value	*Pc*	OR (95% CI)
	%F (N + /n)	F% (N + /n)			
**KIR genotypes**					
*iKIR* > *aKIR*	52.2 (234/448)	64.7 (150/232)	0.002	0.008*	1.67 (1.2–2.32)
*aKIR* > *iKIR*	19.4 (87/448)	13.4 (31/232)			
*iKIR* > 4	67.0 (300/448)	75.0 (174/232)	0.035	0.14*	1.48 (1.036–2.11)
*aKIR* > 4	50.0 (224/448)	40.1 (93/232)	0.015	0.06*	0.66 (0.48–0.92)
**KIR combined genotypes**					
CxTx/*2DS2*	11.1 (50/448)	20.2 (47/232)	0.0074	0.066**	1.84 (1.18–2.88)
CxTx/*2DL2*	12.3 (55/448)	23.7 (55/232)	0.0012	0.011**	2.04 (1.34–3.13)
***iKIR* > *aKIR* carriers subgroup**					
*2DS2* presence	45.2 (105/232)	59.3 (89/150)	0.0087	0.078**	1.76 (1.16–2.67)
*2DL2* presence	49.1 (114/232)	66.6 (100/150)	0.0008	0.0072**	2.05 (1.35–3.17)

*N*+ number of individuals positive for the gene, *n* number of individuals tested for the gene, *HC* healthy control, *OR* odds ratio, *CI* confidence interval. *p* < 0.05: statistically significant; based on two-tailed Fisher’s exact test; *Pc*: corrected *p* values, Pc*: correction factor = 4, Pc**: correction factor = 9.

We next performed a comparative analysis to explore whether simultaneous inheritance of disease risk-related factors influences disease susceptibility (Table 5). We found that patients with lung cancer were more likely to carry the combination of CxTx/*2DS2* compared to controls (20.2% vs. 11.1%, *p* = 0.0074, *P*_*c*_ = 0.066, OR 1.84, CI 1.18–2.88). The likelihood of carrying CxTx/*2DL2* combination was also significantly higher in patients (23.7% vs. 12.3%, *p* = 0.0012, *P*_*c*_ = 0.011, OR 2.04, CI 1.34–3.13). Likewise, disease susceptibility was conferred by the presence of *2DL2* within individuals carrying *iKIRs* > *aKIRs* (66.6% vs. 49.1%, *p* = 0.0008, *P*_*c*_ = 0.0072, OR 2.05, CI 1.35–3.17), this association was weakened in the presence of *2DS2* (59.3% vs. 45.2%, *p* = 0.0087, *P*_*c*_ = 0.078, OR 1.76, CI 1.16–2.67).

## Discussion

In the present study, we assessed the contribution of *KIR* gene content and their corresponding *HLA-I* ligands to lung cancer development in the ethnically homogeneous population of southern Iran. Although previous studies have examined KIRs at genetic, transcriptional, and expression levels in lung cancer, to our knowledge, this is the first report that demonstrates individual *KIR* genes and certain genotypes seem to be associated with susceptibility to lung cancer. Given our sizeable dataset, suggestive interactions between KIR-HLA class I ligands can influence the dynamics of NK cell responses in the lung cancer setting.

Despite existing research addressing the KIR-HLA pair's role in lung cancer, their findings are less consistent. Most recently, in the Chinese Han population, studies conducted by Li et al.^42^ and Yu et al.^43^, found no association between *KIRs* and *KIR*-*HLA* combinations with metastatic NSCLC (mSCLC) and adenocarcinoma, respectively. Consisting with these findings, Wisniewski et al. couldn’t find a significant difference between *KIR* genes or combinations of *KIR*-*HLA* in 269 Polish Caucasians with NSCLC compared with 690 HCs^44^. However, Wisniewski et al. reported carriers of homozygous *HLA*-*C1* and *C2* were more frequent in NSCLC patients, which was not detected in our study^44^. Furthermore, Al Omar et al. observed significantly increased *2DL1*/*C2* and decreased *2DL3*/*C1* in NSCLC patients from England and Northern Ireland^45^. Decreased frequency of *Ile80* allele in NSCLC patients positive for *3DL1*/*Bw4* and decreased *Thr80* allele in SCLC patients positive for *3DS1*/*Bw4,* which is observed by Al Omar et al.^45^, Conforms to our findings of less frequent *3DS1*, *3DL1*/*Bw4* in lung cancer patients. In part, these inconsistent results may be elucidated by the small sample size, heterogeneity of the target population (study population), and divergent distribution of *KIRs* and *HLAs* in different ethnic groups. Importantly, cross-talk of NK cells and the unique microenvironment of each lung cancer subtype^46^ could be responsible for behavioral differences in NK cells, assessing distinct histologic subtypes including (mNSCLC, NSCLC, and SCLC) can presumably lead to conflicting results observed in mentioned studies.

Although we didn’t examine survival rate and response to treatment in lung cancer patients, previous studies obtained interesting results. Yu et al. noted that chemotherapy-treated mNSCLC patients with *KIR2DS4del* and *HLA-Bw4* (*Thr80*) gene expression at the mRNA level exhibited poor overall survival (OS)^43^. Wisniewski et al. reported the striking association of *2DL2*/*2DS2*/*C1* combination with more prolonged survival and better response to therapy in Polish patients^44^, which is discordant with the predisposing effect of *2DL2*/*C1*, *2DS2*/*C2* on lung cancer risk observed in our study. Given the cancer setting, it is crucial to consider the impact of chemotherapy agents on the sensitization of tumor cells to NK cell activity. As it has been shown that stress signals induced by chemotherapy and other treatment modalities can elevate the expression of NK cell-activating ligands^47–49^ or downregulate inhibitory ligands^50,51^, the transient deleterious effect of chemotherapeutic agents on NK cells has also been observed^52,53^.

Our findings primarily determined an association between the carriage of *KIR2DL2* and its activating counterpart *2DS2* with an increased risk of lung cancer. We identified similar results when further analyzing *2DL2* and *2DS2* in the presence of their corresponding *HLA-C1* allele. Consistent with our results, *2DL2* has been demonstrated to confer susceptibility to endometriosis^54^, leukemia^55^, and could be predisposing to lymph node metastasis (LNM) in HNSCC as well^56^. The carrier frequency of *2DL2*/*C1* in malignant melanoma patients with the advanced stage was significantly higher compared with lower-stage patients^57^. Similar results were reported by Naumova et al. showing the association of *2DL2*/*C1* with malignant melanoma^58^. Additionally, *2DL2* and *2DS2* have been shown to confer a predisposition to lymphatic invasion in ER + and PR + breast cancer cases^59^.

According to the “licensing” model, NK cell education via inhibitory receptors integrating with cognate HLA-I ligands endows NK cells with full effector functions and self-tolerance^34^, while NK cell licensing by iKIRs translates into effective sensing of missing HLA I targets, “missing-self” hypothesis, aKIR mediated licensing in the presence of its HLA I ligand induces hypo-responsiveness and renders NK cells impaired responsiveness^60^. Analysis of infiltration pattern and immune cell localization in NSCLC patients revealed that HLA-I negative tumors are predominantly TIL-free and encapsulated by stromal tissue, which consists of a dense structure of FAP + fibroblasts^61^. In addition, reduced TIL infiltration, bigger tumor size, and lymphatic spread have been observed among HLA-I^−^/PD-L1^+^ tumors^62^. To this extent, our finding implies that NK cells of *2DL2*/*C1* carriers are incapable of mounting an efficient response against lung cancer tumors sustaining HLA-I expression due to a defect in “missing-self” recognition. Stromal tissue surrounding tumor lesions, which restrains TILs, including NK cells^61^, could hypothetically represent another immune escape mechanism to avoid NK cell attack in lung cancer with total loss or downregulated HLA-I. It is shown that KIRs recognize altered peptides presented by cognate HLA-I ligands^63^, indicating that alterations in peptide repertoire mostly occurring in the process of tumorigenesis could be detected by KIRs^64^. As a result, stimulation of aKIRs with neoantigens and tumor-inducible ligands expressed on lung cancer cells may prompt cytokine release instead of cytolytic function. Supporting examples would be recent studies in which β_2_-microglobulin–independent ligand has been suggested to be recognized by 2DS2^65^ and 2DS4 interacting with melanoma-derived non-class I MHC proteins^66^. The 2DS2-mediated education in the presence of C1 ligand could raise activation threshold and cause hypo-responsiveness in carriers of *2DS2*/*C1* combination, it also remains possible that upregulated “induced self” ligands mentioned above are unable to overcome such hypo-responsiveness. Due to the tight LD between *2DS2*/*2DL2*, the co-carriage of this combination may exacerbate the detrimental impact attributed to individual genes. More investigation is needed to distinguish the predisposing effect of these two genes on lung cancer.

Moreover, the frequency of *KIR2DS1* and *3DS1* in the HC group was higher than in patients. We couldn’t detect a significant association of *2DS1/C2*, *3DS1/Bw4* combinations with lung cancer, though the *HLA-Bw4 (Ile80)* allele and *KIR3DL1*/*Bw4* were strongly associated with protection against lung cancer. NK cells expressing 2DS1 exhibit an anergy state in individuals carrying *HLA-C2*/*C2*, but not in *HLA-C1*/*Cx* carriers^67^. Likewise, Bw4 (Ile80) could be recognized by 3DS1 positive NK cells derived from donors lacking *Bw4* (*Ile80*), in contrast with NK cells from donors positive for *Bw4* (*Ile80*)^68^. Referring to these studies, it can be suggested that NK cells generated from those *3DS1* and *2DS1* carriers lacking putative *HLA-Bw4* and *HLA*-*C2* ligands have lower activation threshold and might recognize different ligands associated with lung tumor transformation. Therefore, carrying *3DS1* and *2DS1* in the absence of cognate HLA class I ligands could confer better protection against lung cancer. Supporting this explanation, HLA-F open conformers (OCs) have been reported as high-affinity ligands for 3DS1^69^, and Kiani et al. have shown that 3DS1 positive NK cells could be activated upon ligation with HLA-F in which stimulation with HLA-F results in increased antiviral function in NK cells^70^. Interestingly a highly expressed level of HLA-F has been detected in lung cancer^71^, suggesting that HLA-F might be a factor in the association of 3DS1/2DS1 with protection in lung cancer. Furthermore KIRs exhibit various degrees of peptide selectivity, implying that KIRs are sensitive to altered peptides, and this sensory mechanism is more sensitive than “missing self-detection” of lowered HLA-I expression on target cells^72^. Regarding iKIRs, several studies have reported changing peptide repertoire in tumor cells function as peptide antagonism, which down-modulates NK cell inhibition by reducing inhibitory ligands on tumor cells, caused by low-affinity interaction of KIR-HLA^73,74^. The protective effect conferred by *3DL1*/*Bw4* could result from 3DL1 sensitivity to subtle alterations in presented peptides leading to a reduction in inhibitory signals and triggering cytotoxicity in the absence of HLA downregulation.

Estimating the immunological genetic profile could preferably characterize the pathogenesis of lung cancer. Our findings with more frequent T4 gene cluster carriers in HCs, which is likely imparted by the presence of *3DS1*/*2DS1* combination in T4 gene cluster, are in contravention of CxT4 predisposing role in head and neck squamous cell carcinoma and colorectal adenocarcinoma reported in our previous studies of the same population^75,76^. The association of CxTx with lung cancer risk is in line with susceptibility to meningioma reported in CxTx carriers^77^. The results of comparing the different number of *iKIR* and *aKIR* genes, in addition to signifying the strong positive association with lung cancer risk in individuals carrying more inhibitory genes; displayed the influence of activating genes on protection against disease regardless of not being significant after the *p* value correction.

As we emphasized the tight LD between *2DL2* and *2DS2*, it is difficult to dissect the contribution of individual *2DL2* and *2DS2* genes in susceptibility to the disease. Hence, assessing rare genotypes lacking either *2DL2* or *2DS2* is highly informative. Accordingly, we noticed a striking association of the genotype ID19, a rare CxTx genotype carrying *2DL2* in the absence of *2DS2,* with increased lung cancer risk by more than fivefold, suggesting a predominant detrimental impact of *2DL2* over *2DS2*. Regarding our analysis of co-existing susceptibility factors with lung cancer, simultaneous inheritance of CxTx/*2DL2* was shown to predispose carriers to lung cancer. Although, the simultaneous presence of CxTx/*2DS2* did not meet the significant level after *p* value correction and seems to confer a slightly lower risk of lung cancer compared to CxTx/*2DL2*. Superior adverse effect was noticeably related to individuals with *iKIR* > *aKIR* in the presence of *2DL2* rather than *2DS2*. Our findings are strengthened by the report denoting the correlation of higher expression in inhibitory *KIRs* with poor prognosis in lung cancer patients. A higher proportion of NK cells expressing inhibitory KIRs was noticed in NSCLC patients in which lower cytotoxicity and reduced IFN*-*γ production were also shown^78^. Consistently, separate consideration of inhibitory and activating counterparts demonstrated the susceptibility of *2DL1*^+^/*S1*^−^ genotype carriers to cutaneous melanoma and the formation of sentinel lymph node metastasis within individuals with homozygous *HLA*-*C2*^79^. A previous study by Momot et al. suggested that carrying *2DS2*^+^/*L2*^−^ combination is related to a higher risk of scleroderma disease^80^, and similar results were illustrated regarding systemic sclerosis^81^. However, this disagreement with our observations might be due to different mechanisms involved in tumorigenesis and autoimmune disorders. The fact that the difference of genotypes with *iKIRs* > *aKIRs* and CxTx combined with *2DL2* but not *2DS2* remained significant after correction of *p* values implies that these combinations are significantly more strongly associated with lung cancer development. It can also be presumed that the presence of *2DL2* intensifies disease associations.

It is important to highlight the suppressive effects on NK cells suggested to be driven by alveolar macrophages and epithelial lining fluid of the lower respiratory tract^82^. Despite comprising a well-differentiated phenotype, NK cells in lung tissue are exposed to a restricting microenvironment in homeostasis, causing a hypo-functional state of lung NK cells to stimuli in comparison with NK cells of peripheral blood^83,84^. Attenuated killing potency in TME owing to the presence of regulatory T cells (TREGs) and myeloid-derived suppressor cells (MDSCs), limitations of leukocyte infiltration, as well as immunosuppressive factors in lung TME such as adenosine and transforming growth factor β (TGF-β) are yet to be overcome^4,85^. Taken together, it can be speculated that carrying more inhibitory gene content may interfere with NK-mediated immunosurveillance, favoring tumor evasion in the existing suppressor context of lung tissue. On the other end, activating gene content is presumed to restore the functional competence of NK cells, especially in suppressive lung settings.

In conclusion, we report new susceptibility factors and the contribution of *KIR* and *HLA*-*I* encoding genes to lung cancer risk, highlighting an array of genetic effects and disease setting that regulates NK cell responsiveness. Our results suggest that inherited *KIR* genes and *HLA-I* ligands specifying the educational state of NK cells can modify lung cancer risk. In the current study, *HLA*-*I* ligands and co-associations of *KIR*-*HLA* were examined for a limited number of subjects; also an inadequate number of patients with different lung cancer subtypes hindered the evaluation of *KIRs* and *HLA-I* genes impact within subgroups. Thus, larger cohorts assessing the contribution of *KIR*-*HLA* combinations are needed to confirm these associations. Functional analysis might help extend associations to the potential therapeutic strategies against lung cancer. The unique microenvironment of each lung cancer subtype with a varied composition of immune cells is assumed to affect NK cell characteristics^86^. Further investigations on mechanisms involved in NK cell dysfunction in different subtypes might develop into NK-based immunotherapies in lung cancer.

## Materials and methods

### Study subjects

A total of 232 patients with lung cancer (comprising NSCLC: squamous cell carcinoma and adenocarcinoma, SCLC subtypes) and 448 healthy controls (HC) from a homogenous population of the southern part of Iran (Fras province) were included in this case–control study. The enrolled group of 232 unrelated lung cancer patients was made up 85.3% of men and 14.7% of women with a mean age of 64.4 ± 11.1. Healthy controls were comprised 66.5% of men and 33.5% of women with a mean age of 58.13 ± 12.66. The pathologically confirmed lung cancer cases were recruited from Faghihi hospital, Shiraz University of medical sciences. Age and sex-matched HCs with no Family History of Cancer (FHC) were selected from the Motahari clinic. The demographic and clinical characteristics of lung cancer patients were gathered from medical records (Table 1). Due to the fact that smoking status for a very small portion of our lung cancer patients and HCs was available, stratification of the study population based on smoking status wasn’t carried out.

Informed consent from all research participants was obtained, and the study was carried out according to the declaration of Helsinki. This study was reviewed and ethically approved by the Medical Ethics Committee of Shiraz University of Medical Sciences (IR.SUMS.REC.1398.1110).

### KIR genotyping

Genomic DNA extraction from whole blood samples was performed using QIAamp DNA Mini Kit (Qiagen, Germany) as detailed in the manufacturer’s instructions. The PCR-SSP method was used for genotyping 16 *KIR* genes, and *KIR2DS4* variants (*KIR2DS4fl*: *2DS4* full variant, and *KIR2DS4del*: *2DS4* deleted variant) as previously described^87^. Detailed information on primer sequences, thermal conditions, and the mixture of each reaction are reported in our previous study^59^. Reference DNA samples from the UCLA KIR exchange program provided by Prof. Raja Rajalingam were applied to ensure typing accuracy. An alternative SSP-PCR method was used to confirm the unique and unusual KIR genotyping^88^.

The KIR-binding *HLA-A*, *B,* and *C* ligands of 85 lung cancer patients and 178 HCs were typed using the recently developed direct DNA sequencing method. The strategy includes PCR amplifying exons 2 and 3 using *HLA-A*, *B,* or *C* gene-specific primers and direct sequencing of the segment that encodes the KIR-binding region. The process was accomplished in accordance with the method described by Ashouri et al.^89^.

### Data analysis and statistical methods

KIR genotypes of the study participants were assigned according to previous studies^88,90^. The genotype AA comprises fixed gene content (*2DL3-2DL1-2DP1-3DL1-2DS4)* surrounded by frameworks. Carriers of AA genotype-related genes were regarded as homozygous AA, and the remaining subjects were considered as Bx genotype carriers which can be heterozygous AB or homozygous BB. The KIR genotype ID was obtained using the allele frequency database (http://www.allelefrequencies.net/) for all participants.

Based on the linkage disequilibrium, two frequently occurring clusters that include distinct sets of B-haplotype-specific KIR genes have been identified^91^. C4 linkage group comprising *KIR2DS2-2DL2-2DS3-2DL5B* genes is located in the centromeric region of the KIR complex, while the T4 linkage group contains *KIR3DS1*-*2DL5A*-*2DS5*-*2DS1* genes located at the telomeric region of the complex. Concerning the presence or absence of C4 and T4 linkage groups, The Bx genotype carriers were further divided into the following four subsets: C4Tx, CxT4, C4T4, and CxTx^89^. The frequency of C4 and T4 gene clusters were defined by subsequent formulas: C4 = nC4Tx + nC4T4, and T4 = nCxT4 + nC4T4 (n: number of individuals with a particular subset within each group).

The percentage of *KIR* genes in both study groups was indicated by direct counting (number of subjects positive for the gene divided by the number of subjects per population × 100). Differences in the distribution of each *KIR* gene, genotypes, KIR-binding HLA ligands, and KIR-HLA pairs between lung cancer patients and HCs were estimated by two-tailed Fisher Exact probability (*p*) test using SPSS (IBM, US) version 16.0 and Items with *p* < 0.05 were considered as statistically significant. Moreover, the Odds ratio (OR) and 95% Confidence Intervals (CI) were calculated to assess the magnitude of associations. The method expounded by Svejgaard and Ryde^92^ was applied to identify the combined effect of the lung cancer susceptibility factors CxTx^+^/2DL2^+^ and CxTx^+^/2DS2^+^. *p* values regarding the association of genotypes with certain number of genes (*aKIRs* > *iKIRs*, *iKIRs* > *aKIRs*, *aKIRs* > 4, *iKIRs* > 4) were corrected using *P*_n_ = 1 − (1 − *P*)^n^, where n represents the number of comparisons^92^.

### Ethical approval

Ethical approval of the research was confirmed by the Medical Ethics Committee of Shiraz University of Medical Sciences [IR. SUMS.REC.1398.1110].

## Data Availability

The datasets generated and analyzed during the current study are available from the corresponding author upon reasonable request.
